# NMR Characterization and Membrane Interactions of the Loop Region of Kindlin-3 F1 Subdomain

**DOI:** 10.1371/journal.pone.0153501

**Published:** 2016-04-21

**Authors:** Geok-Lin Chua, Suet-Mien Tan, Surajit Bhattacharjya

**Affiliations:** School of Biological Sciences, Nanyang Technological University, 60 Nanyang Drive, Singapore, 637551, Singapore; University of California Berkeley, UNITED STATES

## Abstract

Kindlins-1,2 and 3 are FERM domain-containing cytosolic proteins involved in the activation and regulation of integrin-mediated cell adhesion. Apart from binding to integrin β cytosolic tails, kindlins and the well characterized integrin-activator talin bind membrane phospholipids. The ubiquitin-like F1 sub-domain of the FERM domain of talin contains a short loop that binds to the lipid membrane. By contrast, the F1 sub-domain of kindlins contains a long loop demonstrated binding to the membrane. Here, we report structural characterization and lipid interactions of the 83-residue F1 loop of kindlin-3 using NMR and optical spectroscopy methods. NMR studies demonstrated that the F1 loop of kindlin-3 is globally unfolded but stretches of residues assuming transient helical conformations could be detected in aqueous solution. We mapped membrane binding interactions of the F1 loop with small unilamellar vesicles (SUVs) containing either zwitterionic lipids or negatively charged lipids using ^15^N-^1^H HSQC titrations. These experiments revealed that the F1 loop of kindlin-3 preferentially interacted with the negatively charged SUVs employing almost all of the residues. By contrast, only fewer residues appeared to be interacted with SUVs containing neutral lipids. Further, CD and NMR data suggested stabilization of helical conformations and predominant resonance perturbations of the F1 loop in detergent containing solutions. Conformations of an isolated N-terminal peptide fragment, or EK21, of the F1 loop, containing a poly-Lys sequence motif, important for membrane interactions, were also investigated in detergent solutions. EK21 adopted a rather extended or β-type conformations in complex with negatively charged SDS micelles. To our knowledge, this is the first report describing the conformations and residue-specific interactions of kindlin F1 loop with lipids. These data therefore provide important insights into the interactions of kindlin FERM domain with membrane lipids that contribute toward the integrin activating property.

## Introduction

Kindlins are a small family of band four-point-one, ezrin, radixin, moesin (FERM)-containing cytoplasmic proteins that regulates integrin-mediated cell-cell and cell-ECM adhesion [1]. Kindlin-1, -2 and -3 have tissue-specific expression pattern. Kindlin-1 is expressed primarily in epithelial cells and kindlin-2 is ubiquitously expressed, whereas kindlin-3 is expressed in hematopoietic and endothelial cells [2–5]. They have non-redundant functions and their biological significance underscored by debilitating diseases. The Kindler syndrome is characterized by skin fragility and atrophy as a consequence of mutation(s) in kindlin-1 that disrupt epithelial cell adhesion [2,6–8]. Mutation(s) in kindlin-3 that leads to defective leukocyte and platelet adhesive properties have been shown to be the molecular basis of the disease Leukocyte Adhesion Deficiency (LAD) type III, in which patients exhibit bleeding disorder and have a compromised immune system [9–12]. Aberrant kindlin-3 expression has been reported in different cancers [13,14]. Kindlin-2 gene ablation in mice is embryonic lethal [15]. Apart from myogenesis, hemostasis, and chrondogenesis, kindlin-2 is involved in cancer metastasis [16–20].

**Fig 1:**
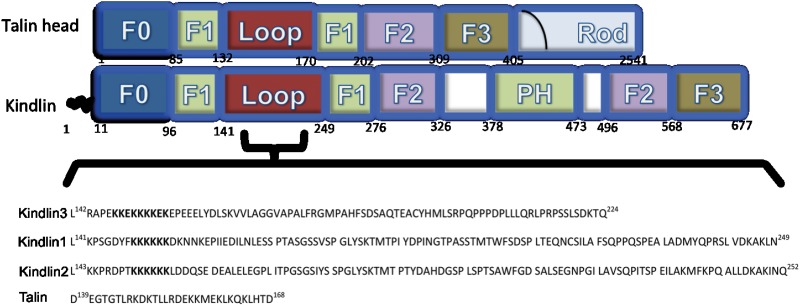
Domain organization of talin and kindlin and primary structures of the F1 loops. The FERM domain of talin, also called talin head, and kindlin is sub-divided into several subdomains F0, F1, F2 and F3. The FERM domain of kindlin also contains a PH domain inserted within the F2 subdomain. The full-length talin also contains a rod domain which is absent in kindlins. The F1 domains of talin and kindlin contain a loop insert for membrane interactions. The primary structures of F1 loop of kindlin-1, kindlin-2 and kindlin-3 and talin are shown.

The three kindlin paralogs share a similar domain organization. Each kindlin is composed of subdomains, F0 from the N-terminal followed by F1, F2 and F3. The F2 subdomain is bisected by a pleckstrin homology (PH) domain [21] (Fig 1). The F3 subdomain contains a phosphotyrosine binding domain (PTB) that binds to the membrane distal NxxY/F motif in the cytoplasmic tail of the integrin β1, β2, and β3 chains [9,10]. The PH domain within the F2 subdomain is involved in phosphoinositide binding [22–25]. The F0 domain of kindlin-1 adopts an ubiquitin-like fold and it is required to localize kindlin-1 to focal adhesions [26]. A notable feature of the F1 subdomain shared by all three kindlins is the presence of a long loop that contains a stretch of poly-lysine sequence. A similar loop, although much shorter in length, is found in the F1 subdomain of talin head region, but it has a propensity to form a helical conformation and interacts with negatively charged lipid vesicles [27]. The F1 loop of kindlin-1 binds to negatively charged lipid vesicles, as revealed from co-sedimentation assays [28]. However, structural characterization and membrane interactions of the F1 loop of kindlin-2 and kindlin-3 are lacking. In this study, we used NMR and optical spectroscopic methods to investigate the conformations of the F1 loop region (residue Leu142-Gln224) (Fig 1) of kindlin-3 in aqueous solution. NMR ^15^N-^1^H HSQC titration experiments were performed to demonstrate residue specific interactions of F1 loop region with liposomes containing zwitterionic lipid POPC, negatively charged lipids POPS or POPG. We also examined the conformational characteristics of a peptide fragment (residues E146-K166) which corresponds to the lysine-rich sequence of the F1 loop. The current study explains membrane specific association of kindlin-3 by its large loop region, a key requirement for the activation of integrins by co-activator kindlins. Further, conformations and membrane interactions of the loop region of kindlin-3 showed significant differences in comparison to the F1 loop region of talin. These differences may contribute in part to the distinct properties of kindlin and talin in integrin activation.

## Materials and Methods

### Expression, isotope (^15^N, ^13^C) labeling and purification of kindlin-3 F1 loop

The expression, labeling and purification of kindlin-3 F1 loop was performed at the Protein Production Platform, School of Biological Sciences, Nanyang Technological University, Singapore. In brief, kindlin-3 cDNA that encodes Leu142-Gln224 was PCR cloned into expression plasmid pNIC28-Bsa4 (Addgene, Cambridge, MA) that contains an N-terminal 6His-TEV site. The expression plasmid was transformed into BL21(DE3) Rosetta T1R and large scale culture was performed using the LEX system (Harbinger Biotech). 5 mL of TB media was inoculated with transformed BL21(DE3) glycerol stock supplemented with kanamycin and cultured for 24 h at 37°C with shaking at 200 rpm. Bacteria were pelleted by centrifugation at 1500 g for 10 min. Cell pellet was used to inoculate 1.5 L of labelled M9 minimal media, containing either ^15^N ammonium sulfate or ^15^N ammonium sulfate and ^13^C glucose, supplemented with antibiotic overnight at 37°C with shaking at 200 rpm. Protein production was induced by 0.5 mM isopropyl-β-D-thiogalactopyranoside (IPTG) at 18°C overnight when the bacteria culture reached an OD600 value of 0.6. Cells were pelleted and re-suspended in lysis buffer (100 mM HEPES, 500 mM NaCl, 10 mM Imidazole, 10% glycerol, 0.5 mM TCEP, pH 8.0) containing benzonase (1000 U) (Merck, Darmstadt, Germany) and protease inhibitor cocktail (Calbiochem, Merck-Millipore group) followed by sonication on ice. Kindlin-3 F1 loop was purified using affinity chromatography on a Ni-NTA superflow (Qiagen) column followed by size exclusion chromatography on a HiLoad 16/60 Superdex 75 prep grade column on a FPLC system.

### Circular dichroism (CD) studies

The full-length F1 loop or EK21 peptide was reconstituted in 50 mM sodium phosphate buffer (pH 5.8) containing 150 mM NaCl and 20 mM β-ME. The CD absorbance of the F1 loop (100 μM) or in the presence of 50 mM dodecylphosphocholine (DPC) (Avanti) or 50 mM dodium-dodecyl sulfate (SDS) (Merck) was measured in a 0.2 mm path length cuvette (Hellma, Germany), from 240 nm to 190 nm using a Chirascan CD spectrophotometer (Applied Photophysics, UK). CD data were recorded at 1 nm time step of 1s. Three spectra were recorded for the three different conditions (F1 loop in free solution, in DPC and in SDS solutions) and were later averaged and subtracted from the corresponding background buffer contributions and converted to mean residue ellipticity.

### Preparation of Small Unilamellar Vesicles (SUVs)

SUVs were prepared as previously described with modifications [27]. 20 mg of POPG, POPS or POPC was dissolved in 2 mL of chloroform and freeze dried overnight at -80°C. The lipid film was then rehydrated in 20 mM HEPES buffer (pH 5.8) containing 150 mM NaCl and 2 mM TCEP that was pre-heated 50°C. The rehydrated lipid film was incubated for 1 h at50°C in a water bath followed by 5 freeze-thaw cycles. The sample was sonicated for 1 h at 37°C in a water bath. The resulting lipid solution was passed through a 50 nm membrane using a lipid extruder (Avanti).

### NMR experiments of F1 loop of kindlin-3 and EK21 peptide

All NMR spectra were recorded on a Bruker DRX 600-MHz instrument equipped with an actively shielded cryoprobe and pulse-field gradients. Spectra were processed using Topspin 2.0 (Bruker) and analyzed with Sparky and CCPN programs. Backbone resonance assignments of 500 μM double-labeled F1 loop dissolved in 50 mM sodium phosphate buffer (pH 5.8) containing 150 mM NaCl and 20 mM β-ME were obtained by performing a set of triple resonance experiments CBCA(CO)NH and HNCACB. ^15^N-edited 3-D NOESY-HSQC experiment (mixing time 200 ms) was carried out for ^15^N-labelled sample in the above mentioned buffer. 200 μM of ^15^N-labelled F1 loop dissolved in 50 mM sodium phosphate buffer (pH 5.8) containing 150 mM NaCl and 20 mM β-ME was titrated with DPC (Avanti) and SDS (Merck) at concentrations of 50 and 100 mM. 200 μM of ^15^N-labelled F1 loop dissolved in 20 mM HEPES buffer (pH 5.8) containing 150 mM NaCl and 2 mM TCEP was titrated with POPG, POPS or POPC vesicles at concentrations of 2, 4 and 6 mM. ^15^N-^1^H HSQC spectra were recorded for the free protein and for each titration points for the 3 different kinds of vesicles. Percentage signal attenuation from the HSQC spectral peaks intensity was calculated for residues following this expression, % signal attenuation = (I_o_-I)/I ×100, where Io and I represent ^15^N-^1^H HSQC peak intensity of F3 loop either in free solution or in the presence of SUVs, respectively.

The EK21 peptide (500 μM) dissolved in aqueous solution containing perdeuterated SDS (CIL, USA) was analyzed by two-dimensional ^1^H-^1^H TOCSY and NOESY experiments, with 60 ms and 200 ms mixing times, respectively, at 35°C.

## Results

### Backbone resonance assignment and secondary chemical shifts of the kindlin-3 F1 loop

**Fig 2:**
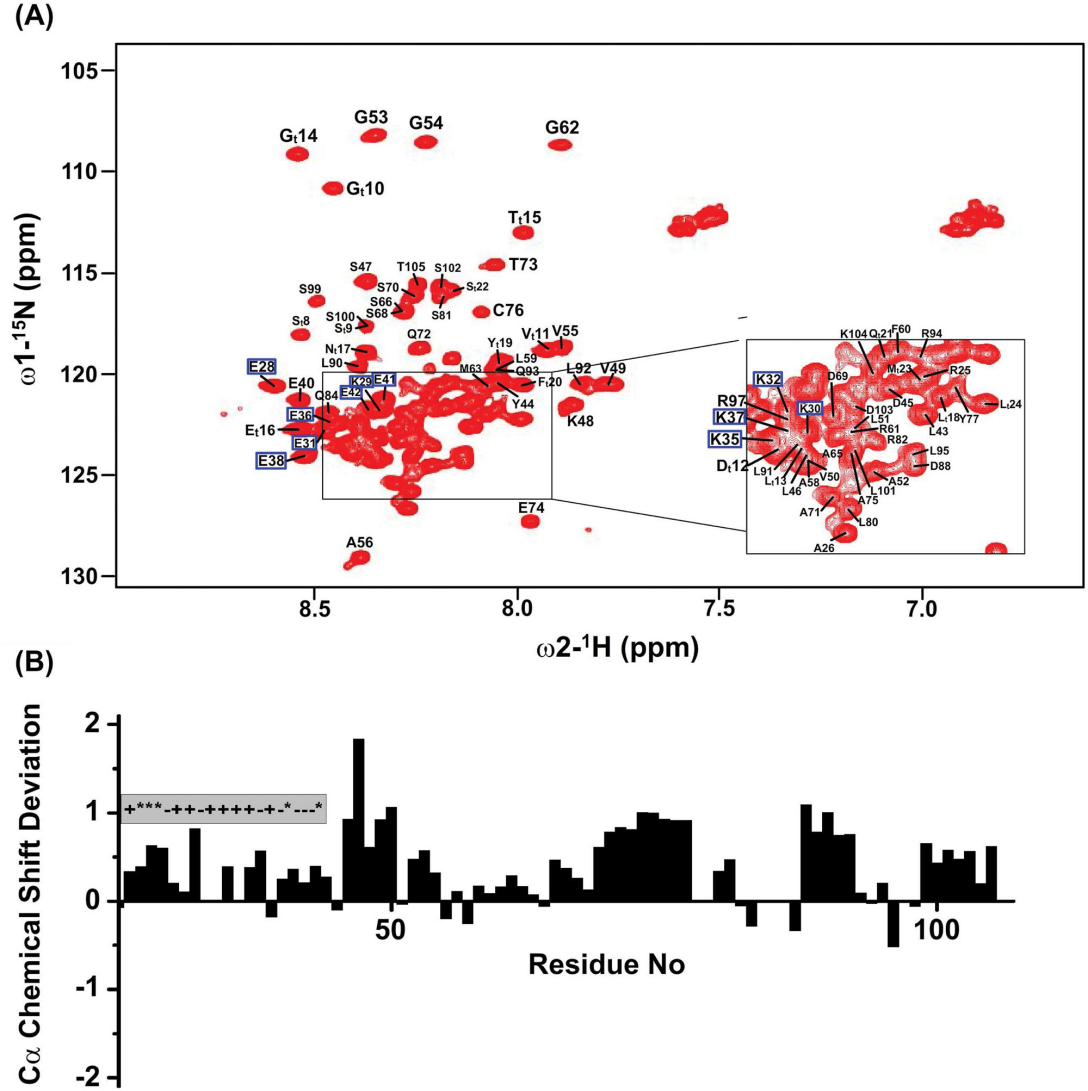
NMR characterization and secondary structures of the F1 loop of kindlin-3. (panel A) Two-dimensional ^15^N-^1^H HSQC spectrum of 83-residue long F1 loop of kindlin-3 showing resonance assignments of ^15^N and amide proton single bind correlations. The F1 loop of kindlin-3 contains residues L142-Q224, but numbered here as L1-Q83. HSQC cross-peaks arising from the additional N-terminal tag residues are marked as ‘t’. (panel B) Secondary chemical shift of ^13^Cα chemical shift for individual residues of F1 loop of kindlin-3.

**Fig 3:**
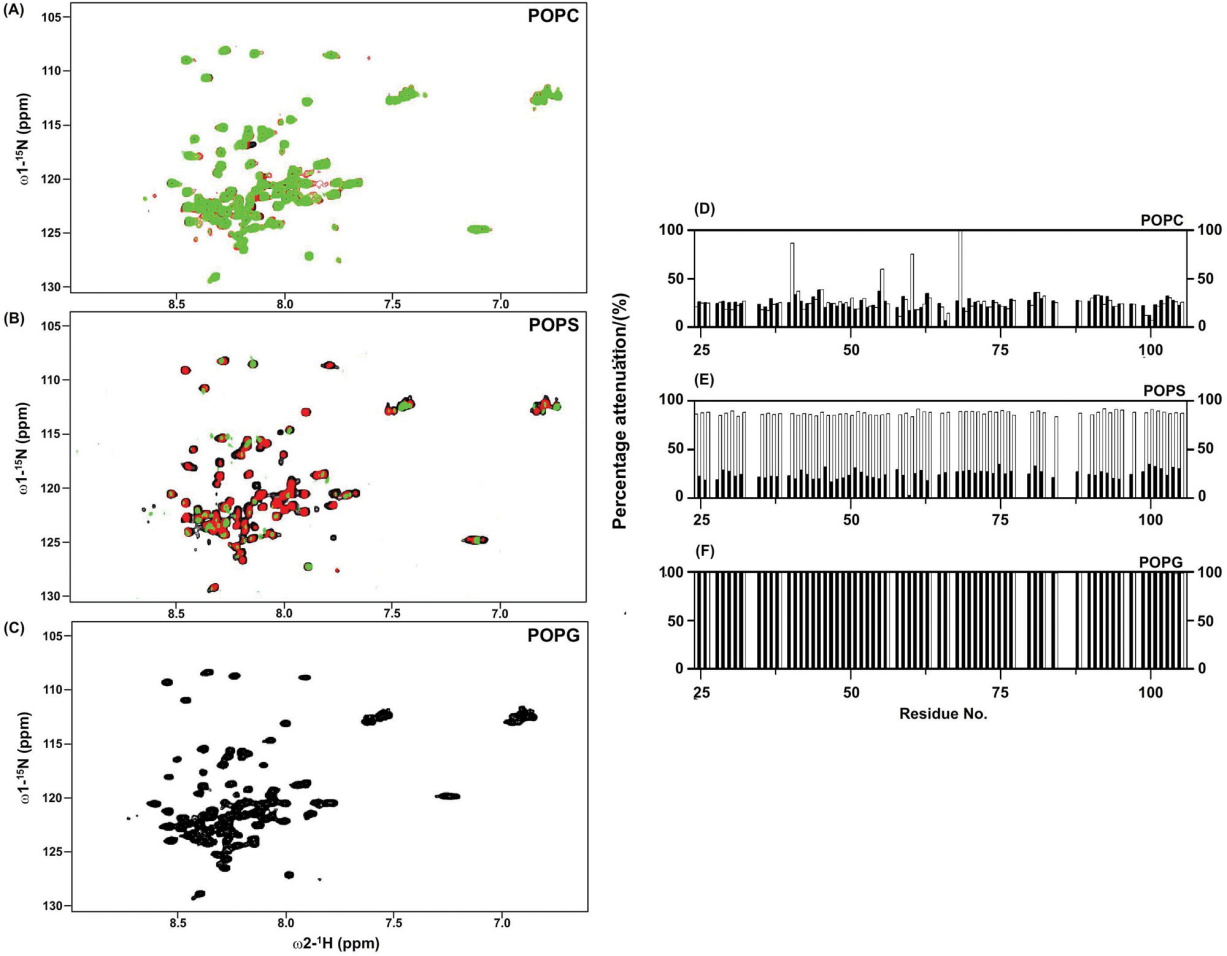
Lipid specific interactions of SUVs with the F1 loop of kindlin-3. Overlay of ^15^N-^1^H HSQC spectra of F1 loop at different concentrations, 0 mM (black contour), 4 mM (red contour) and 6 mM, of POPC (panel A), POPS (panel B) and POPG (panel C) SUVs. Bar diagrams showing attenuations of ^15^N-^1^H HSQC cross-peak intensity of F1 loop of kindlin-3 after additions of 2 mM (black bar) and 4 mM (white bar) of POPC (panel D), POPS (panel E) and POPG (panel F) SUVs, respectively.

The ^15^N-^1^H HSQC spectrum of the 83-residue loop region of kindlin-3 showed limited chemical shift dispersion, ~ 0.8 ppm, of the amide proton resonances, whereas ^15^N chemical shifts of the amino acids were better dispersed (Fig 2A). These spectral features are typically observed for natively unfolded proteins and denatured proteins. The amino acid sequences of the natively unfolded proteins are characterized by a low sequence complexity with high percentage of polar and charged amino acids [29, 30]. The primary sequence of the kindlin-3 F1 loop is characterized by over 65% polar and charged amino acids with several repetitive segments (Fig 1). The N- and C-termini of the loop contains a repetitive Lys and Glu rich sequence motif and a Pro/Leu rich sequence, respectively (Fig 1). Backbone (^1^H, ^15^N, ^13^Cα) and sidechain ^13^Cβ resonances were assigned by combined analyses triple resonance HNCACB and CBCA(CO)NH experiments. Backbone resonance assignments of the loop region were achieved for over 95% non-Pro residues including Glu/Lys repetitive sequence motif at the N-terminus (Fig 2A). Secondary ^13^Cα chemical shifts and 3-D NOESY-HSQC spectrum of the F1 loop were analyzed to determine conformational characteristics. Deviation of ^13^Cα chemical shift from random coil or the secondary chemical shift is a sensitive indicator of the secondary structures of proteins [31]. Secondary chemical shift values presented after sequence dependent corrections were made [32]. Well populated or stably folded helical and β-sheet structures would show either a positive deviation or a negative deviation, respectively, of >1 ppm in secondary chemical shifts. The ^13^Cα secondary chemical shift of the loop region of kindlin-3 showed that most of the residues experienced a positive deviation with occasional break by negative secondary chemical shift (Fig 2B). However, the secondary chemical shift values were restricted and predominantly lower than 1 ppm. In particular, the stretch of N-terminal poly-Lys residues i.e. R2-D22, including residues L28-S45 and C-terminal residues Q70-T82 showed lowest secondary chemical shift deviation (Fig 3). The secondary shift of residues P60-P63 (P60QPP63) could not be determined because of the presence of multiple Pro residues. Note, there are stretches of residues that showed positive secondary shift close to or more than 1 ppm such as residues L23-V27 (LSKVV) and residues D65-L69 (DPLLL) (Fig 2). Further, analyses of the 3-D NOESY-HSQC spectrum of the F1 loop revealed the presence of only sequential and intra-residue NOE connectives (data not shown). The NOE pattern also suggests an absence of stably folded conformations of the loop region in aqueous solution.

### Interactions of the loop region of kindlin-3 with SUVs

A series of ^15^N-^1^H HSQC spectra of kindlin-3 loop in the presence of SUVs containing POPC, POPS or POPG at different concentrations (2 mM, 4 mM and 6 mM) were acquired. Fig 3 (panels A-C) shows overlay of ^15^N-^1^H HSQC spectra of kindlin-3 loop in free solution (black contour) and in the presence of 4 mM (red contour) and 6 mM (green contour) SUVs. As can be seen, no discernable changes in ^15^N-^1^H HSQC spectra of kindlin-3 loop were observed upon inclusions of POPC SUVs (Fig 3A). Whereas, ^15^N-^1^H HSQC spectra were found to be more perturbed, in terms of disappearance of cross peaks and loss of signal intensity, at 6 mM POPS vesicles compared to 4 mM POPS (Fig 3B). By contrast, ^15^N-^1^H HSQC spectra of kindlin-3 loop showed dramatic signal attenuation both at 4 mM and 6 mM POPG SUVs (Fig 3C). ^15^N-^1^H HSQC signal attenuations of kindlin-3 loop were estimated at 2 mM and 4 mM concentrations of SUVs. Most of the residues, except for few, of kindlin-3 loop delineated limited signal attenuation in the presence of POPC SUVs (Fig 3D). Residues E17, V32, F37 and S45 of kindlin-3 loop appeared to be perturbed in 4 mM POPC (Fig 3D). Signal attenuation of the residues of kindlin-3 loop was less conspicuous at 2 mM POPS, however, most of the residues experienced 70 to 80% signal loss at 4 mM POPS (Fig 3E). By contrast, almost all the residues of kindlin-3 loop demonstrated remarkably higher signal attenuation, ~100% even at 2 mM POPG concentration (Fig 3F). Thus, the aforementioned data indicated that the kindlin-3 loop preferentially interacts with the negatively charged lipid bilayers, whereas loop/membrane interactions are limited for the neutral lipids.

### Interactions of kindlin-3 loop region with detergent micelles

**Fig 4:**
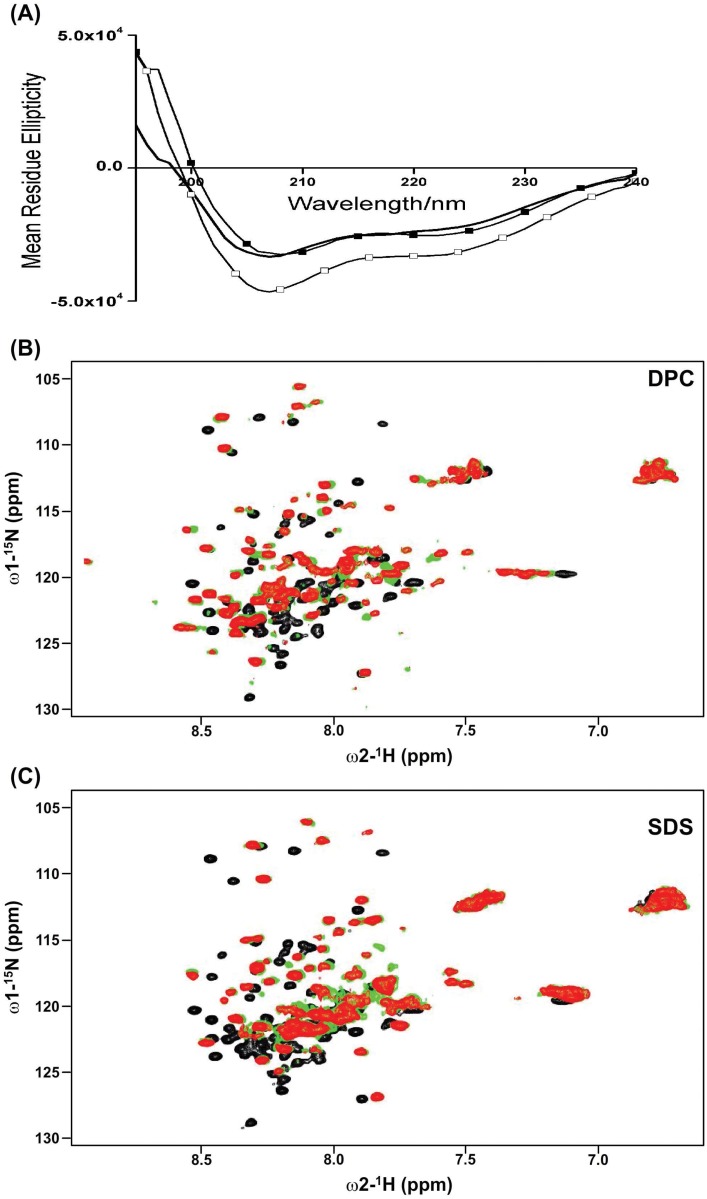
Secondary conformations and micelle interactions of the F1 loop of kindlin-3. (panel A) Far UV-CD spectra of F1 loop of kindlin-3 in aqueous buffer solution (in solid black line), in solutions containing 50 mM DPC (in black squares) and 50 mM SDS (in white squares). (panel B) Overlay of ^15^N-^1^H HSQC spectra of the F1 loop of kinldin-3 in aqueous buffer solution (black contour), in solutions containing either in 50 mM (red contour) or 100 mM (green contour) DPC. (panel C) Overlay of ^15^N-^1^H HSQC spectra of the F1 loop of kinldin-3 in aqueous buffer solution (black contour), in solutions containing either in 50 mM (red contour) or 100 mM (green contour) SDS.

In order to assess interactions and conformational characteristics of the loop region in membrane mimic environments, far UV CD and ^15^N-^1^H HSQC spectra were obtained in solutions containing negatively charged SDS and zwitterionic DPC micelles. UV CD spectra (240–190 nm) of the kindlin-3 loop in detergent free solutions and in solutions containing 50 mM detergents showed a relatively intense band at 208 nm and a less pronounced band around 222–225 nm (Fig 4A). However, these CD bands were found to be somewhat more intense in solutions containing SDS micelles (Fig 4A). Observation of a shallow CD band at 222/225 nm would indicate that the loop region may assume dynamic populations of partly folded or nascent helical conformations both in aqueous solution and in DPC detergent. In other words, the kindlin-3 loop does not undergo any significant conformational changes in zwitterionic DPC detergent solutions. The presence of more intense CD band at 222 nm in SDS detergent solutions is suggestive of stabilization of such incipient helical conformations. Interactions of the loop region of kindlin-3 were further investigated by acquiring ^15^N-^1^H HSQC spectra in different concentrations of DPC and SDS detergent solutions. Compared with kindlin-3 loop region in free solutions, significant changes in ^15^N and ^1^H chemical shifts were detected in solutions containing either DPC (Fig 4B) or SDS micelles (Fig 4C). From the ^15^N-^1^H HSQC spectra of kindlin-3 loop in detergent solutions, the disappearance and broadening of a number of resonances were observed. ^15^N-^1^H HSQC spectral changes were also more extensive in the presence of SDS detergent solution. We have acquired 3-D NMR spectra for backbone resonance assignment and structural characterization of kindlin-3 loop in SDS and DPC solutions. However, overlapping resonances and broader line width precluded NMR structural characterization of the full length loop in detergent solutions.

### Conformational characterization of the N-terminal poly-Lys peptide fragment of the loop region

**Fig 5:**
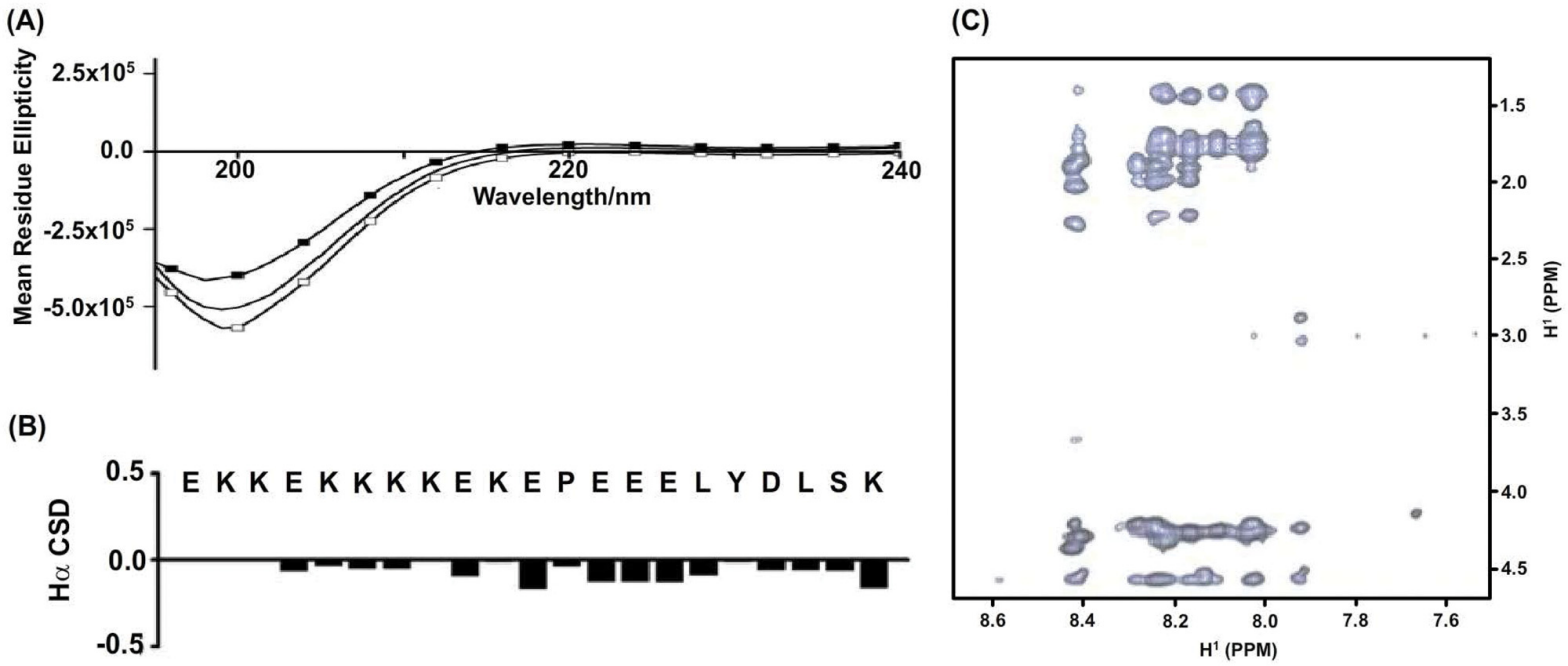
Conformational characteristics of EK21 peptide fragment derived from N-terminus of the F1 loop. (panel A) Far UV CD spectra of EK21 peptide in free buffer solution (solid line) and in solutions containing either 50 mM DPC (solid line with black squares) or 50 mM SDS (solid line with white squares). (penal B) Bar diagram showing secondary chemical shifts of αH resonances of residues of EK21 in solutions containing SDS micelles. (panel C) Selected section of ^1^H-^1^H two-dimensional NOESY spectrum of EK21 peptide obtained in aqueous solutions containing 200 mM perdeuterated SDS.

The N-terminus of the loop region contains a Lys-rich sequence that has been implicated in membrane interactions of the kindlin-3 [28]. We therefore examined the conformation of a 21-residue long synthetic peptide or EK21 (EEKKEKKKKEKEPEEELYDLSK) which corresponds to the Lys-rich sequence in the kindlin-3 loop. Far UV CD spectra of EK21 were obtained in free solution and in 100 mM DPC or SDS solutions (Fig 5A). CD spectra of EK21 were predominantly characterized by the presence of a single minima centered at 200 nm either in aqueous or in detergent solutions. Such CD band has been predominantly observed for proteins and peptides assuming extended or random coil-like conformations. Hence, the EK21 peptide fragment appeared to be lacking well defined secondary structures even in the presence of detergent. To better determine the conformational characteristics of ER21 peptide, two-dimensional ^1^H-^1^H NMR, TOCSY and NOESY, experiments were performed in solutions containing 200 mM perdeuterated SDS. Sequence-specific resonance assignments of EK21 were achieved by combined analyses of TOCSY and NOESY. Secondary chemical shift of Hα resonances of EK21 showed limited deviation from respective random coil values, potentially indicating absence of discernable folded secondary structures (Fig 5B). (Fig 5B). Selected sections of NOESY spectra of EK21 in SDS micelles showing NOE connectivity from the low-field shifted amide and aromatic proton resonances (along the w2 dimension) with up-field shifted aliphatic proton resonances (along the w1 dimension) are shown (Fig 5C). There was a limited number of NOE cross-peaks correlating backbone/backbone and backbone/sidechain proton resonances in the NOESY spectra of EK21 (Fig 5C). The observed NOEs were found to be predominantly sequential or intra-residue in nature. Therefore, the paucity of diagnostic NOE contacts for EK21 peptide in detergent solutions indicates the absence of populated folded structures in the membrane mimicking environments.

## Discussion

### Conformations and interactions of kindlin-3 loop with lipid vesicles

Talins and kindlins regulate the activation of integrins via the interactions of their FERM domain with the integrin β cytoplasmic tail. [33–35]. The FERM domain of talin and kindlin contains sub-domains, namely F0, F1, F2 and F3. The F3 subdomains of talin and kindlin bind to the membrane proximal and membrane distal regions in the integrin β tail, respectively, which are key steps in the process of integrin activation [36–39]. In addition, recent studies have demonstrated that the full-activation of integrins require interactions of effector proteins including talins and kindlins with the phospholipid membrane [21, 28]. Therefore, the formation of multi-protein complexes at the membrane surface is critical in the functional regulation of integrins. Exposed cationic surfaces belonging to the folded sub-domains of FERM, e.g. F2 and F3 of talin, F0 of kindlin, have been shown to be involved in membrane binding [40, 41]. Kindlins also contain a PH domain that binds membrane phosphoinositides [42]. Apart from the above mentioned membrane interactions, initial studies have demonstrated that a 30-residue long loop of the F1 sub-domain of talin is important for the activation of integrin [27]. The talin F1 loop is rich in cationic residues and assumes segments of amphipathic helical structures in the presence of helix-stabilizing co-solvent, 2,2,2 tri-fluoroethanol [27]. These cationic residues are found to be occupying one face of the amphipathic helical structure and critical for binding to acidic phospholipids vesicles [27]. The long F1 loops of kindlins bear no apparent sequence similarity with the F1 loops of talins. In particular, unlike the F1 loop of talin that contains distributed cationic residues, the kindlin F1 loop displays a conserved cluster of cationic residues at its N-terminus (Fig 1). Functional studies with kindilin-1 showed that F1 loop is required for focal adhesion targeting and optimal activation of αIIbβ3 integrin potentially through its interactions with acidic phospholipids of membranes [28]. Further, the poly-Lys (KKKKKK) motif of the kindlin-1 loop appeared to be necessary for binding to acidic lipids POPS and co-activation of αIIβ3 integrin [28]. In order to gain better insights of F1 inserts of kindlins, here, we have investigated conformations; liposome and detergent binding of the F1 loop of kindlin-3 comprised the shortest loop among kindlins. NMR data, ^15^N-^1^H HSQC, 3-D NOESY and secondary chemical shifts, of the F1 insert of kindlin-3 showed that while the 83-residue long globally unfolded in aqueous solution but contains regions of partially folded helical segments. In particular, residues L23-V27 (LSKVV) and residues D65-L69 (DPLLL) showed intrinsic tendency to assume a single turn helical conformations. The intrinsic helical conformations of D65-L69 region may be occurring due to the helical conformational preferences of multiple Leu residues. Populated incipient helical conformations of residues L23-V27 could be arising due to potential non-polar packing interactions among alkyl sidechains of residues L23/V26 and/or L23/V27. It may be noted that residue Val in proteins has been known to promote β-sheet structures; however, helical conformations have been determined for Val rich protein including surfactant-associated polypeptide [43]. These dynamic helical conformations of the kindlin-3 loop are likely to gain further stabilization upon binding to negatively charged lipid membranes. In order to understand residue-specific interactions of the F1 loop with membrane, we prepared SUVs consisted of lipids containing different charge head groups and titrated in a series of ^15^N-^1^H HSQC experiments (Fig 3, panels A-C). We find that the F1 loop of kindlin-3 delineated diminution of intensity of HSQC cross-peaks upon additions lipid vesicles (Fig 3, panels D-F). The ^15^N-^1^H HSQC resonances of F1 loop of kinldin-3 demonstrated least perturbation in the presence of POPC SUVs containing a neutral head group (Fig 3, panels A and D). Interestingly, resonances of residues E17, V32, F37 and S45 were found to be more perturbed compared to other residues of the F1 loop (Fig 3D). These observations indicated that most part of the F1 loop of kindlin-3 lacks significant interactions with zwitterionic lipid vesicles. However, transient interactions with zwitterionic PC lipid vesicles may occur for residues e.g. V32, F37 and S45, located in the hydrophobic stretch V^26^VLAGGVAPALFRGMPAHFS^45^ of the F1 loop of kindlin-3. Remarkably higher resonance intensity changes of ^15^N-^1^H HSQC spectra of F1 loop were detected in titrations with SUVs of POPS and POPG containing negatively charged head groups, suggesting stronger lipid-protein interactions. However, POPS and POPG showed a different concentration dependent signal diminutions of HSQC spectra. Intensity of ^15^N-^1^H HSQC cross-peaks of almost all the residues were highly attenuated even in the presence of 2 mM POPG SUVs (Fig 3, panels C and F), however, under similar conditions POPS SUVs have yielded much lower reduction of cross-peak intensity (Fig 3 panels B and E). These observations suggest the POPS lipid SUV may confer a somewhat lower affinity interactions, compared to POPG lipid SUV, with the F1 loop of kindlin-3. Notably, HSQC signals of the F1 loop were dramatically attenuated at higher concentrations, 4 or 6 mM, of POPS vesicles, indicating binding interactions. Most notably, almost all of the residues of the F1 loop of kindlin-3 have demonstrated resonance perturbations in the presence of negatively charged SUVs. These observations strongly indicated that most of the residues of the F1 loop could be closely associated with the negatively charged SUVs. By contrast, NMR studies showed that only the C-terminus residues among the 30-residue F1 loop of talin interacted with the negatively charged lipid vesicles [27]. The F1 loop of kindlin-1 also demonstrated preferential interactions with negatively charged POPS vesicles compared to POPC vesicles as revealed by co-sedimentation experiments [28]. Most importantly, current results showed that the F1 loop of kindlin-3 binds to the negatively charged lipids employing most of the residues including the poly-Lys residues at its N-terminus. Whereas the F1 loop of kindlin-3 delineated transient interactions with zwitterionic lipids involving fewer residues located at the middle hydrophobic sequence, it should be noted that the plasma membrane of mammalian cells is rich in neutral PC like lipids. We surmise that such transient interactions may help in recruiting kindlin to the plasma membrane followed by stronger membrane binding utilizing the negatively charged lipids.

### Conformational characterization of the F1 loop and its N-terminal peptide fragment in detergent micelles

Solutions containing fast tumbling detergent micelles e.g. SDS and DPC, have been frequently utilized to determine structures of integral membrane proteins and also membrane interacting peptides and proteins by NMR methods. The F1 loop of kindlin-3 showed interactions with negatively charged SDS and zwitterionic DPC micelles as shown by the conspicuous changes of ^15^N and NH chemical shifts (Fig 4, panels B and C). Detailed conformational characterization of the full-length F1 loop was not possible at this stage due to unfavorable line-width and overlapping resonances. Hence, we assessed the global conformations of the F1 loop in detergent micelle solutions by CD spectroscopy. CD studies showed that the F1 loop of kindlin-3 undergoes structural stabilization of helical conformations in SDS micelle solution (Fig 4A). On the other hand, conformations of the F1 loop appeared to be more flexible either in free solution or in solutions of DPC micelles. The N-terminal region of the F1 loop of all kindlins is characterized by the presence of multiple cationic residues including a continuous stretch of four to five Lys residues. Previous functional studies revealed that the N-terminal region is critically involved in lipid interactions and activation of platelet integrin [28]. In the full length 83-residue F1 loop, this cationic N-terminal region appears to be lacking discernable folded or helical conformations in free solutions, as evident from NMR data (Fig 2B). In order to gain more structural information, we investigated a peptide fragment or EK21 encompassing the N-terminus poly-lys motif. CD spectra of EK21 peptide showed a single band ~ 200 nm suggesting lack of defined secondary structures in solutions containing negatively charged SDS micelles (Fig 5A). Further, observations of fewer diagnostic NOEs (Fig 5C) and limited secondary chemical shifts (Fig 5B) of EK21 in SDS micelles indicated extended conformations of EK21 in SDS micelle solution. Therefore, unlike F1 loop of talin that forms helical conformations in membrane mimic solvent, the N-terminal ploy-lysine region of the F1 loop of kindlin-3, although important for membrane interactions, remains in an extended conformations in the membrane mimic environment.

**Fig 6:**
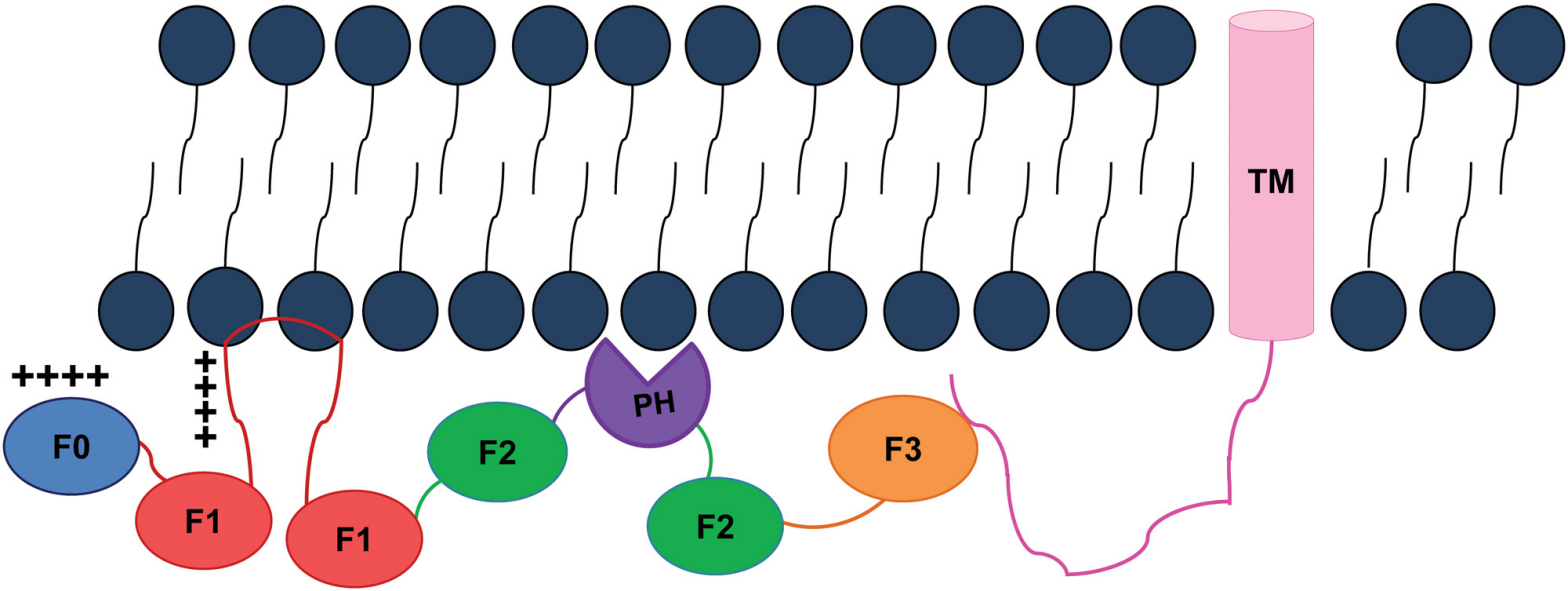
Localization of the FERM domain of kindlin in membrane. A hypothetical cartoon showing proximity of the FERM domain of kindlin to the lipid membrane and interactions with the beta cytosolic tail. The FERM domain interacts with the phospholipid lipid bilayer (shown as black stick with round head) through F0 subdomain (in blue), PH (in purple) and potentially with split F2 (in green) and F3 (in pink) subdomains by ionic interactions with exposed positively charged surface. The unfolded F1 loop of the F1 subdomain (in red) might insert into the lipid membrane using N-terminal positive charged residues of poly-lys and sidechain of hydrophobic residues. Such a membrane localization of the FERM domain of kindlin might bring the F3 subdomain for optimal interactions with the beta cytosolic tail attached with its TM (in light purple) for activation of integrins.

In conclusion, the F1 loops of kindlins and talins are critically involved in activation mechanism of integrins through their interactions with the acidic phospholipids of membranes. In this work, we demonstrated that the F1 loop of kindlin-3 preferentially interacted with acidic phospholipids. The F1 loop of kindlin-3 has also displayed transient interactions with the neutral PC lipid. The F1 loop of kindlin-3 showed remarkable differences in terms of conformations and membrane association with that of the F1 loop of talin. Such conformational and lipid embedding differences between the F1 loops of kindlin and talin may be implicated in the functional specificity as observed from loop swapping [28]. Most of the residues of the F1 loop of kindlin-3 sustained interactions with the negatively charged phospholipids. We surmise that the functional orientation of the FERM domain of kindlin would perhaps require a larger membrane association through its F0 domain, PH domain and the F1 loop region (Fig 6). The proposition would require investigations employing full length kindlin, integrins and lipid bilayer. Regardless, membrane anchoring of the FERM domain of kindlin might aid in stabilizing interactions with the distal part of the β cytosolic tail of integrin. Further, it is possible that the membrane tethering of the FERM domains of kindlins and talins facilitates their simultaneous interactions with high affinity to the integrin β cytosolic tail, leading to the separation of the integrin α/β cytoplasmic tails required for integrin activation [1, 33, 44].
